# The Dynamics of Nanoparticle-enhanced Fluid Displacement in Porous Media - A Pore-scale Study

**DOI:** 10.1038/s41598-018-29569-2

**Published:** 2018-07-24

**Authors:** Tannaz Pak, Nathaly Lopes Archilha, Iara Frangiotti Mantovani, Anderson Camargo Moreira, Ian B. Butler

**Affiliations:** 1 Teesside University, Middlesbrough, UK; 2 Brazilian Synchrotron Light Laboratory (LNLS), Brazilian Center for Research in Energy and Materials (CNPEM), Zip Code 13083-970, Campinas, Sao Paulo, Brazil; 3 University of Santa Catarina, Florianópolis, Brazil; 4 University of Edinburgh, Edinburgh, UK

## Abstract

This work provides new insights into the dynamics of silica nanoparticle-based removal of organic fluids (here oil) from naturally occurring porous media. We have used 4D (time-resolved 3D) imaging at pore-scale using X-ray computed micro-tomography (μCT) technique. The captured 3D tomographic time-series data reveal the dynamics of immiscible oil displacement from a carbonate rock upon injection of nanoparticle (NP) suspensions (0.06 and 0.12 wt% SiO_2_ in deionised water). Our analysis shows significant pore-scale remobilisation of initially trapped oil upon injection of the NP suspensions, specifically, at higher concentration. Our data shows that oil clusters become significantly smaller with larger fluid/fluid interface as a result of the higher concentration NP injection. This paper demonstrates that use of 2D radiograms collected during fluid injections allows monitoring flow dynamics at time resolutions down to a few seconds using conventional laboratory-based μCT scanners. Here, as an underlying mechanism for oil remobilisation, we present the first 4D evidence of *in-situ* formation of an oil in water emulsion induced by nanoparticles.

## Introduction

Developing a detailed understanding of the pore-scale dynamics of multiphase fluid flow in porous material is important in a wide range of applications, including: (i) remediation of groundwater resources contaminated by non-aqueous phase liquids (NAPL), (ii) oil recovery from hydrocarbon reservoirs, and (iii) secure storage of CO_2_ in geological formations through carbon capture and storage technology. Specifically, in processes (i) and (ii) the aim is to remobilise the organic fluid, already trapped in porous media, and to achieve enhanced recovery/removal efficiency. Conversely, in the case of (iii) the aim is to ensure capillary trapping of supercritical CO_2_ within porous rocks for the medium to long term. Fluid entrapment and remobilisation is governed by the balance between the capillary and viscous forces (measured by capillary number, Nc) which is in turn a function of porous media structure, its wetting preference, flow velocity, and fluid/fluid interfacial tension (IFT)^1^.

Wettability is a fluid-fluid-solid property which reflects the preference of the solid surface to be in contact with a fluid in presence of a second fluid^2^. Preferentially water-wet systems form most groundwater resources as well as a significant proportion of hydrocarbon reservoirs. A residing non-wetting fluid (e.g. oil) can be displaced from the host porous media by injection of the wetting fluid (e.g. water). Such an immiscible displacement, however, can be an inefficient process that may leave behind a significant proportion of the original oil. Pore-scale imaging studies have revealed that oil forms disconnected clusters which may occupy a single pore or span across many connected pores. It may be surrounded, partially or completely, by water films^3–8^. This trapped oil is the target of enhanced oil recovery (EOR) processes in oil industry^9^ and remediation processes in environmental engineering^10^. Among other technologies, the use of surfactants has been extensively researched at laboratory, pilot and field scales. More recently, the injection of water-based nanoparticle (NP) suspensions has received attention as a recovery enhancement technique. While surfactant injection into geological sites has become a commonly practiced EOR/remediation method, the injection of NP suspensions is still at its early stages^11^. Recent developments in production and engineering of NPs at industrial scale^12–14^ has made them readily available as an alternative to surfactants^15^. Trapped oil can be (i) displaced from porous media in the presence of non-reactive NPs such as silica^16,17^, or (ii) degraded *in-situ* using metal (such as zerovalent iron) NPs^18,19^. For the former the main mechanisms identified in the literature to date are: (i) reduction of fluid-fluid IFT^20^, and (ii) wettability alteration from oil-wet to water-wet or mixed-wet systems^21^. Among the non-reactive NPs studied in the literature (including Al_2_O_3_, TiO_2_, ZnO, Fe_2_O_3_, and SiO_2_) silica NPs are shown to perform better than other particles in terms of increasing oil recovery from rocks^22^. In addition, silica NPs are known to be the particle of choice for a broad range of industrial applications, including biotechnology^23^ and nanomedicine^24^. Their wide application motivates investigation of the suitability of this particle for groundwater remediation/enhanced oil recovery.

Among the primary challenges of injecting nanofluids (i.e. NP suspensions) in porous media are (i) ensuring the particles remain in suspension throughout the process^25^, and (ii) predicting/controlling the particle retention in porous media during transportation^26^. Given their small sizes (<100 nm) compared to typical pore-throat sizes of many naturally occurring geological formations (conventionally micro-meter and larger), NPs should cause no clogging issues if properly suspended^27^. In practice these challenges need to be dealt with on a case-by-case basis.

In this contribution, we utilise μCT imaging to investigate the use of NPs to enhance oil remobilisation within a heterogeneous carbonate rock. For the first time, we present direct visualisation of NP-induced oil remobilisation at pore-scale.

## Results

**Figure 1:**
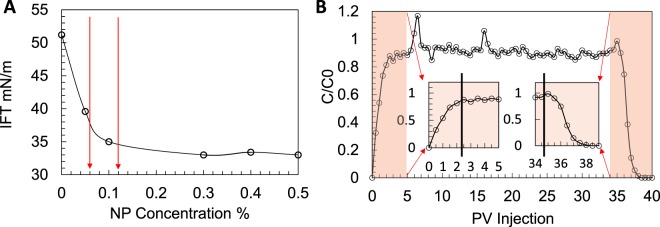
(**A**) IFT (oil/nanofluid) as a function of NP concentration, the red arrows show the two NP concentrations used in this study, (**B**) breakthrough curve.

In order to design an optimised NP-enhanced oil removal process we studied the (i) nanofluid/oil IFT, (ii) particle retention for this NP/rock pair, and (iii) NP suspension stability over time. For the chosen NP (hydrophilic silica, 30 nm) the nanofluid/oil IFT decreases with increase of NP concentration, see Fig. 1A. The rock under study is water-wet. Particle retention needs to be measured to examine the suitability of the selected particle for the rock. For this the breakthrough curve is developed that plots the nanofluid concentration in the effluent (normalised by the injecting nanofluid concentration) against the injected nanofluid volume (measured in pore-volume, PV), see Fig. 1B. For the selected NP/rock pair the effluent contains close to 90% of the injected NPs after injecting 2.5 PVs of nanofluid. At this point the NP concentration in the effluent has plateaued. Also, the NP concentration drops in the same fashion in the subsequent water flush, i.e. at PVs 35 to 37.5. This behaviour shows a limited retention for the selected NP/rock pair, allowing the particles to stay within the suspension making them available to act at the oil/nanofluid interface. In addition to selection of the NP type breakthrough curves inform selection of slug size (i.e. the number of PVs of nanofluid required for the process to be effective). Here we used 3 PVs for each NP injection step.

The selected NP displays stable suspensions at the chosen concentrations within the first week (specifically the first two days) after preparation, see Fig. S1.

The two-phase flow experiment included a primary drainage (oil displacing water), an imbibition (water displacing oil, waterflooding), and two subsequent nanofluid injections at concentrations of 0.06 wt% and 0.12 wt%. The detailed methodology is presented in section 5. The μCT image acquisition was a two-component process: (i) at the end of each injection step a 3D volumetric image of the rock was captured, and (ii) 2D radiographic images were collected during fluid injections. The latter were collected since the time-scale of fluid mobilisation is significantly faster than that of laboratory 3D data acquisition. The imaging details are presented in section 5.

### Analysis of Oil Remobilisation based on 3D Volumetric Images

**Figure 2:**
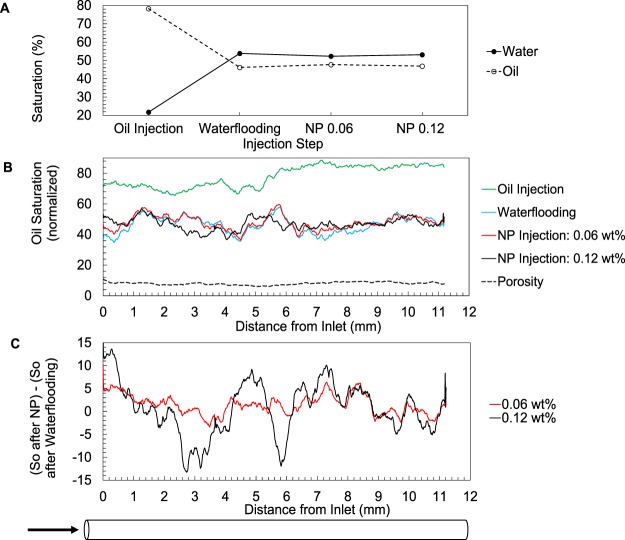
(**A**) Water and oil saturation at the end of each injection step. (**B**) Oil saturation profile along the core calculated based on 3D µCT volumes. The separation between the curves shows oil remobilisation, more pronounced during the 0.12 wt% NP injection. (**C**) Profile of oil saturation change (with reference to the waterflooding) caused by the two nanofluid injection steps. More oil remobilisation occurred at 0.12 wt%.

Figure 2A shows fluid saturations calculated based on the imaged 3D volumes. An initial oil saturation of 78.28% was reduced to 41.02% by the water injection (Fig. 2A). The nanofluid injections did not result in a decrease in the end point oil saturation within the studied volume, yet the saturation profiles along the core length (Fig. 2B) show that local fluid distributions have changed as a result of nanofluid injections. In other words, the previously trapped oil (at the end of waterflooding) has been remobilised upon injection of the nanofluids. The oil saturation profile displays a more pronounced change for the higher concentration nanofluid injection, see Fig. 2C. See Figs S2 and S3 for example 2D slices and 3D renderings of the oil phase after these injection steps.

**Figure 3:**
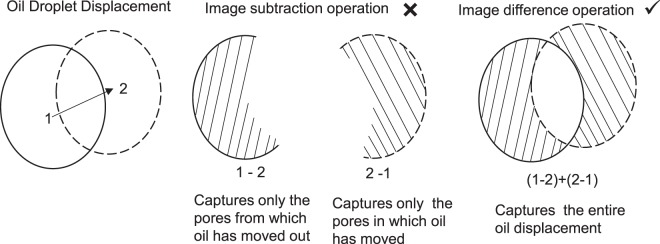
Image difference operation (right) better represents the oil displacement compared to image subtraction operation.

In order to understand the oil remobilisation we use the image difference operation (as explained in Fig. 3) which captures the entire displacement unlike simple image subtraction operation which only partly captures the displacement. In Fig. 3 an example oil droplet is displaced from point 1 to point 2. This displacement is only partly captured by subtracted images (i.e. 2-1 or 1-2), whereas the difference image, i.e. (2-1) + (1-2) provides a more effective representation of this displacement.

**Figure 4:**
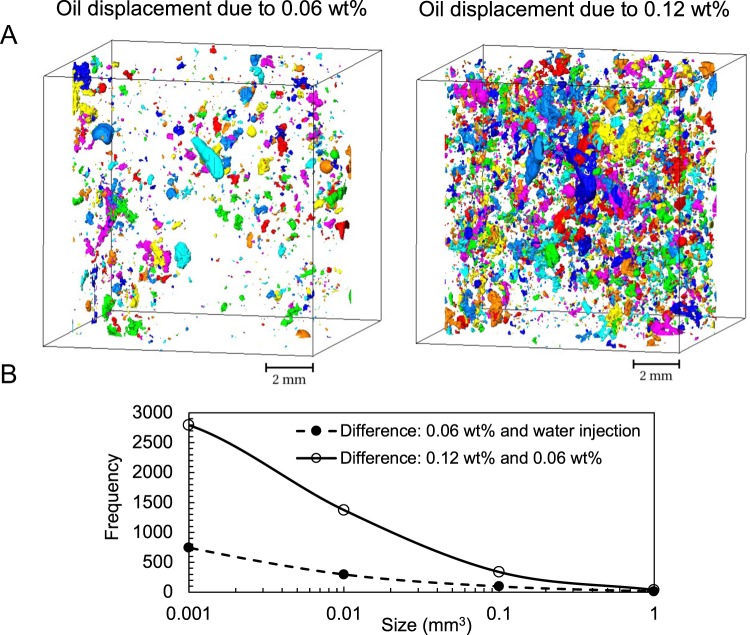
Use of image difference operation to study the oil remobilisation (**A**) 3D rendering of the oil displacement as a result of NP injections at 0.06 wt% (left) and 0.12 wt% (right), (**B**) Size distribution of the displaced oil.

Using the above-mentioned image difference operation Fig. 4 shows 3D visualisations and quantitative analysis of the extent of oil displacement as a result of NP injection steps. In Fig. 4A the image difference is calculated for (i) waterflooding and 0.06 wt% NP injection (left image), and (ii) the two NP injections (right image). Figure 4B shows the size distribution of the objects shown in 4A (i.e. the displaced oil droplets). It is clear that significantly more (i.e. an increase of 2 to 4 times in each bin) oil displacement has occurred as a result of NP injection at 0.12 wt%.

### Analysis of Oil Remobilisation based on 2D Radiograms

**Figure 5:**
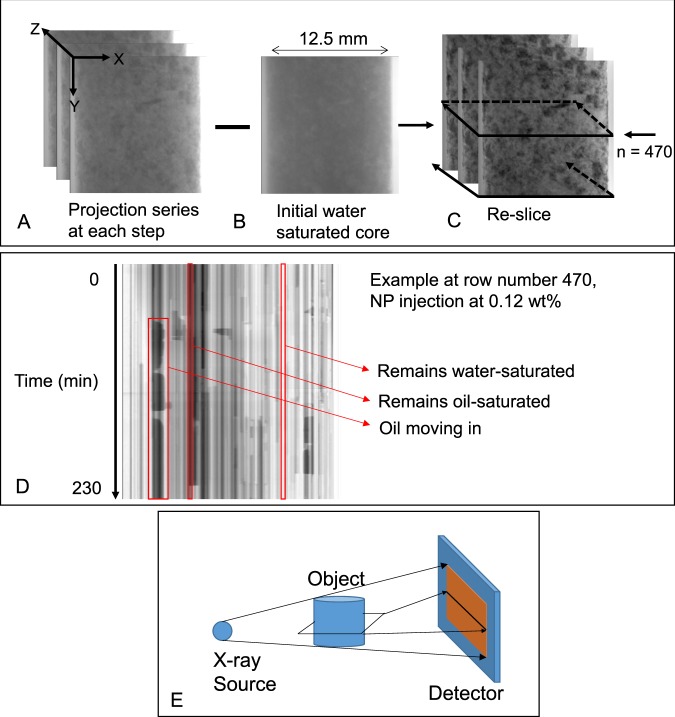
Subtraction of the radiogram captured at initial conditions (**B**) from a stack of radiograms captured during different injection steps (**A**). Reslicing in Z (time) direction as shown in (**C**) creates a new stack of 2D images for which an example is shown in (**D**). (**E**) Each row on the radiograms is a 1D projection of a 2D cross sectional slice of the sample.

To analyse the collected 2D radiograms an image captured at the initial state (Fig. 5B) was removed from all stacks (Fig. 5A). This removes the rock and enhances the fluid/fluid contrast. In order to monitor fluid movements into/within/out of the field of view each stack of radiograms was then resliced in the Z direction as shown in Fig. 5C. Since each stack is collected over time, here the Z dimension of the image stack represents time. Such a reslicing operation looks at the signal detected by each row of the camera over time, this results in a new 2D image for each row. Each row on the radiograms is a 1D projection of a 2D cross sectional slice of the sample (Fig. 5E).

An example resliced image is shown in Fig. 5D. This particular image shows the fluid movements at row number 470 from the bottom of the detector during 0.12 wt% NP injection. The darker phase is the oil phase (which was doped) and the brighter phase is the aqueous fluid. On a resliced image, looking along the time dimension (i.e. vertical lines) pixels that display little change in greyscale represent minimum local fluid movement over time, while oil/water movements are evident from sudden changes in greyscale values. In this example, oil movement within this portion of the rock is evident by droplet like objects highlighted by the left red box.

**Figure 6:**
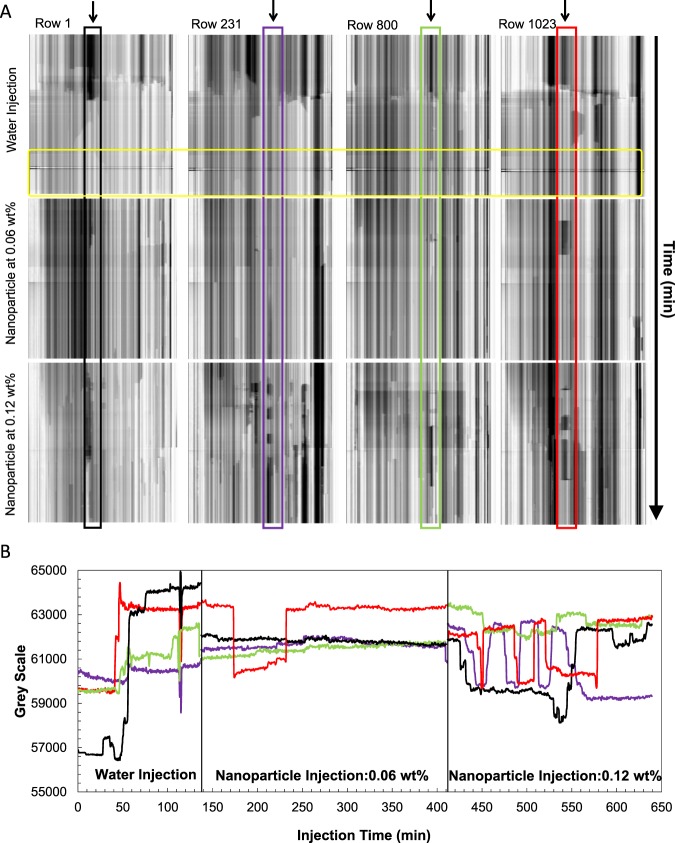
(**A**) Resliced radiograms that reflect the fluid movements at these four points of the core. (**B**) Line profiles plotted at the example points indicated with the highlighted boxes in (**A**), Black: row 1, purple: row 231, green: row 800, and red: row 1023. The yellow box shows the last ~100 minutes of the water injection step.

Here we further discuss the pore-scale dynamics that occur during the water and the two subsequent NP injection steps by looking at the resliced radiograms at the four rows of 1, 231, 800, and 1023 (Fig. 6). Rows 1 and 1023 show the inlet/outlet of the imaged section, respectively. Vertical line profiles at the points marked by the arrows in Fig. 6A are plotted in Fig. 6B.

The initial water injection step shows a clear displacement of oil by water represented by the signal jump from darker to brighter greyscales in Fig. 6B. In the last ~100 minutes of this step (Fig. 6A, yellow box) little fluid displacement seems to happen suggesting that the remaining oil is trapped by capillary forces. The resliced 2D radiograms display significant fluid movements during the 0.12 wt% NP injection step. The line profiles presented in Fig. 6B confirm what is seen in Fig. 2C, i.e. more pronounced oil displacement during the 0.12 wt% NP injection compared to the previous step. These profiles are plotted for the points indicated within the coloured boxes on Fig. 6A. The images show smaller oil objects (droplets) reflecting a more dynamic oil remobilisation during the 0.12 wt% NP injection step. The oil movements into and out of the imaged section of the core causes the end point oil saturation (calculated based on the 3D images) to shows little change (Fig. 2A) despite the evident oil remobilisation during the 0.12 wt% NP injection.

### Pore-scale Dynamics of Oil Remobilisation based on 3D Volumetric Images - First evidence for ***in-situ*** formation of an oil-in-water emulsion induced by NPs

**Figure 7:**
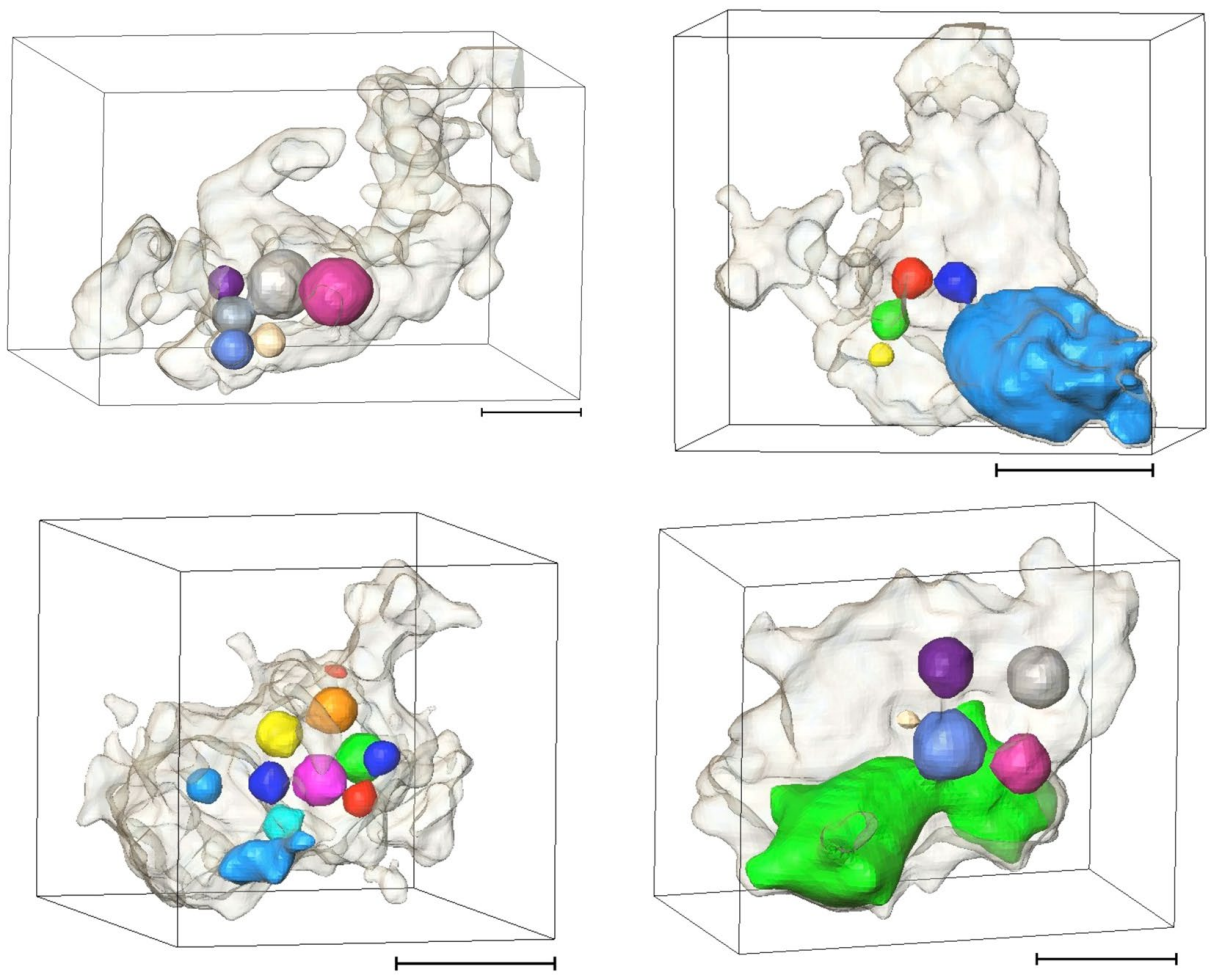
3D renderings of ow emulsion captured after the 0.12 wt% NP injection, scale bar is 0.3 mm. The droplets are the oil phase and the shadow is the pore confining them. Example µCT slices through these pores are presented in Fig. S3.

Here we present the first evidence on the presence of an *in-situ* oil-in-water (ow) emulsion during NP-based oil remobilisation. Figure 7 shows example emulsions that were observed after injection of the 0.12 wt% nanofluid. The presence of emulsion was not captured after the 0.06 wt% NP injection.

Although these images are collected only after the injections were completed the captured emulsion phase shows clear re-structuring and remobilisation of the oil that was previously trapped in the pore system. Figure S3 shows other example 2D µCT slices of emulsions found confined within the pore-space of this rock after injection of the 0.12 wt% nanofluid.

**Figure 8:**
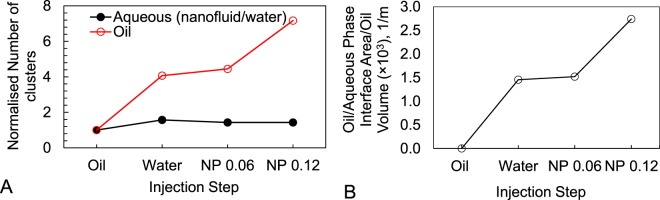
(**A**) Normalised number of fluid clusters, and (**B**) fluid/fluid interface area at different injection steps.

The emulsion is mainly observed within the larger pores, with equivalent diameters ranging from 0.4 to 1.6 mm. These pores are distributed across the imaged section of the core. In total, the oil clusters showed a sharp rise in number (7 fold) as a result of the 0.12 wt% NP injection, while aqueous phase displays little change, i.e. being the wetting phase, it remains mostly connected throughout the experiment (Fig. 8A). In this Figure the cluster numbers are normalised against the number of clusters after the initial oil injection, therefore, both series start from the value of 1. The fluid/fluid interface area also shows a more profound increase as a result of the 0.12 wt% nanofluid injection (Fig. 8B), respectively, 4.5% and 80% after 0.06 wt% and 0.12 wt% NP injections.

## Discussion

Adsorption of NPs to the fluid/fluid interface causes the nanofluid/oil IFT to decrease with increasing NP concentration^20,28^. The trend is explained by reduction in total interfacial energy as fluid/solid contact surfaces have lower energy levels compared to fluid/fluid interfaces^29^. This means the particles act as surfactants^29^. Within the context of remobilisation of a trapped oil from porous media, a reduced IFT is favourable since it reduces the capillary forces, and hence eases the oil removal.

Our images do not capture a decrease in the end point oil saturation, within the imaged volume, as a result of the nanofluid injections. This is due to the limited field of view which only allowed imaging the middle part of the core. Here, we rather focus on understanding the pore-scale dynamics of the NP injections, and the changes of the trapped oil structure as a result of NP injections. Closer investigation of the 2D radiograms/3D volumetric images captured during/at the end of each injection reveals that significant pore-scale dynamics occur as a result of nanofluid injection. Specifically, this remobilisation is more pronounced at the higher concentration which is in agreement with the profiles shown on Fig. 2C. 3D images captured at the end of each injection step show the remaining oil clusters are smaller in size providing larger fluid/fluid interface area after the second NP injection step. Our images provide a quantitative measure of the change in structure of the remaining oil (Fig. 8) which is important for design of subsequent oil displacement/*in-situ* degradation processes.

The size of the objects captured on the resliced 2D radiograms represent a combination of the amount of displaced oil and its residence time at that particular slice. Here, this residence time refers to the amount of time an oil droplet spends in a particular pore or at a pore-throat before (if) it is remobilised. Therefore, longer objects represent displacement of a larger volume of oil and/or a longer period of time spent by a droplet at that pore. It has been shown that the events that occur in a pore are impacted by those that occur within neighbouring pores such as during Haines jumps and cooperative pore-body filling events^3,30^. Therefore, direct statistical analysis of the amount and size distribution of such droplet-like objects may not reflect the saturation or droplet size distributions. Rather, comparing such resliced images for different fluid injection processes (i.e. water, NPs) can reflect the amount of fluid trapping instability that is induced by the NPs, i.e. the pore-scale dynamics of the system are captured.

We suggest that the pore-by-pore oil displacement during the nanofluid injection is affected by the following two mechanisms: (i) *oil droplet formation* at the pore-throats^31^ due to reduced IFTs caused by having NPs in the system, (ii) *stabilisation of the generated emulsion* caused by adsorption of the NPs at the oil/nanofluid interface, which prevents droplet coalescence^29^. The emulsion type is controlled by the wetting preference of the particles in use. Here hydrophilic silica particles with no coating were used and hence an ow emulsion is formed. The observed emulsions are consistent with Pickering type^32^ ow emulsions that have well known industrial application such as pharmaceutics, drug delivery, cosmetics, and food industry^33^. Formation of an ow emulsion is specifically useful where the target oil is highly viscous as ow emulsions have lower viscosities (compared to oil), this improves oil mobility^34^.

Both visual inspection and quantitative analysis of these images show the droplets forming these emulsions are of a broad sizes-range which could potentially be correlated with (i) the pore/pore-throat size distribution, and (ii) the remaining space within the pore-body at the point of droplet formation. Another potential scenario is that droplets could have formed within different-size pores prior to being displaced to the pores hosting them at the point of capturing the 3D images. This data set does not allow understanding such details as, due to limited time-resolution, the pore-throat(s) through which the oil displacements have occurred cannot be identified. Also, in many cases the pore-throat sizes are close to the image resolution which limits reliable extraction of their size distribution. Fast synchrotron-based imaging, however, may provide the required data with sufficient time-resolution to correlate the emulsion structure with pore structure and NP concentration in future investigations. The latter’s control on the IFT impacts the oil droplet sizes that grow at each pore-throat before they snap-off.

It is worth noting that the pore-scale mechanisms behind formation of the observed emulsions are different from the droplet fragmentation mechanism^4^ as (i) the emulsions are not observed within the pores initially hosting larger oil droplets trapped at the end of the water flooding stage, (ii) the NP injection steps were designed to take place within the capillary dominated flow regime (Nc~10^−7^) similar to the waterflooding injection.

Collectively, visual observations and quantitative analysis of μCT images show that the NP-induced oil remobilisation was more effective at the second NP injection step (higher concentration of 0.12 wt%) while the first NP injection step (lower concentration of 0.06 wt%) made little difference. These observations suggest the limited particle retention (within the first three PVs as shown in Fig. 1) could have made the lower concentration NP injection less effective. Therefore, when designing an effective NP-based oil recovery process it is critical to study the required concentration/slug size (different for different systems of NPs/fluid/rock). 4D experiments using μCT imaging provide an excellent means to fine tune the design of nanotechnology-based oil recovery processes.

## Conclusions

Using X-ray μCT we imaged the pore-scale dynamics of oil removal of from a carbonate rock specifically as a result of injecting silica-based nanofluids at two concentrations of 0.06 wt% and 0.12 wt%. The rock studied is a naturally occurring and heterogeneous rock in comparison with simple pore-systems often investigated in laboratory-based studies reported in the literature.

Our observations and analysis reveal that nanofluid injection can successfully remobilise oil phase previously trapped within porous media. We demonstrated that 4D imaging studies allow direct measurement of the changes in the structure of the oil phase as a result of NP injections. Smaller oil clusters and larger fluid/fluid interface is the main outcome of NP injection at its more effective concentration, i.e. here the 0.12 wt%.

For the first time, we demonstrate a sequence of 2D radiograms captured during the injection processes, when resliced in the time dimension, can provide valuable insight into the dynamics of oil remobilisation at high temporal resolution (seconds). This is indeed very useful for studies conducted using laboratory-based μCT scanners which are limited by their slow scanning rates.

Here we present direct evidence on *in-situ* formation of an oil-in-water emulsion, caused by NP-induced reduction in IFT, and stabilised by the presence of NPs. Emulsion was observed globally in the core and within a wide range of pore-sizes, but mainly the larger ones.

## Materials and Methods

### Fluid Preparation and the Rock Sample

The nanofluids were prepared by diluting a concentrated aqueous suspension of bare silica nanoparticles (by US-Nano, 30 nm) in 18.2 MΩ.m deionized water. We used a mineral oil (50% 1-iododecane and 50% dodecane). Iododecane works as a dopant that increases the X-ray attenuation. The selected oil mixture enhances image contrast and matches the density of the fluids. ρ_o_ = 1.005 g/cm^3^ is very close to ρ_w_ = 1 g/cm^3^ and that of the two nanofluids (1.0006, 1.0012 g/cm^3^). This eliminated the potential for gravity-driven fluid redistribution during 3D image acquisition.

The rock is a North American outcrop, Silurian dolomite from the Thornton Formation. The vuggy porosity of this rock allowed capturing almost 70% of the pore space by these µCT images. The core used (D = 12.8 mm, L = 34.5 mm) had a measured total porosity of 19.1%. µCT imaging captured a porosity of 13.3%, making 30% of the pore-space unresolved due to the limited resolution (13.25 µm). The PV of this core is 850 µL. The dolomite under study displays a complex and multi-scale pore structure. Analysis of 3D μCT images of this rock acquired at different length-scales shows the pore-sizes range from sub-micron to mm scales^35^. Mercury porosimetry data shows that this rock has a wide range of pore-throat sizes spanning from 1–200 μm, see Fig. S4.

### Test Procedure

Particle retention was measured by nanofluid (0.3 wt%) injection in a water-saturated core plug of this rock, a miscible displacement process, while effluent was sampled. For the two-phase displacement experiments the core was initially vacuum-saturated with water and flushed with water after vacuum saturation to exclude air. Oil injection (i.e. primary drainage) was followed which achieved an initial oil saturation of 78%, after 10 PVs, with no further production of water. Next, deionised water was injected to mimic waterflooding that is a common improved oil recovery technique. The subsequent two steps involved injection of nanofluids at 0.06 wt% and 0.12 wt% concentration. The experimental set-up is shown in Fig. S5. All injections were performed under capillary dominant flow regime (Nc~10^−7^) to mimic the processes pertinent to field-scale operations. Back pressure was kept at 518 KPa during the nanofluid injections. Fluid injections were performed from the bottom of the core. Details of flow rates and the injected volumes are described in Table S1.

### Imaging

The images were captured using a laboratory based µCT scanner which meant the 3D imaging was only possible at the end of each injection step. To monitor processes that occur during fluid injections we constantly collected 2D radiograms during injections (3000–5000 radiograms, every 2 seconds). A 2D radiogram of a 3D object represents the cumulative attenuation of the incident beam projected on a flat detector. The local fluid movements within the core were captured on these 2D radiograms. It is not possible to extract pore-by-pore analysis based on these 2D images due to the lack of the third dimension.

At least one 3D image was collected after each injection, this was made possible using an X-ray transparent fluid flow cell^36^, integrated with the μCT instrument built at the University of Edinburgh. Each scan is comprised of 2000 radiograms (exposure time: 1.5S) captured during a full 360° rotation. The source-sample and sample-detector distances were 37 mm and 549 mm, respectively. A 0.8 mm aluminium filter was used to reduce the measurement noise and beam hardening effect. The X-ray source voltage and current were 120 kV and 195 µA, respectively.

Our µCT images captured 11 mm length of core 2–3 mm away from the inlet face of the core. Hence, the field of view of the captured images do not show the inlet and production faces of the core. This section was chosen to ensure that capillary end effects do not influence the volume investigated. In a similar experiment on the same rock (but a different plug) capillary end effect was measured to affect a zone of 15 mm close to the core outlet^37^.

UV spectrometry (wavelength 228 nm) was used to generate the breakthrough curve, the readings showed ±1% error (Fig. S1A). UV scattering response shows a linear increase with NP concentration for the nanofluids used in this study indicating stability, see Fig. S1 in the supplementary information. This NP suspension stability was studied by measuring the UV scattering response (wavelength: 268 nm) for nanofluids over time. The results are presented in Fig. S1B.

Image processing and quantifications were performed using (i) 3D images: Avizo software version 9, (ii) 2D radiograms: ImageJ version 1.51. Prior to quantitative image analysis of tomographic data, the measurement noise was suppressed using the non-local means filter^38^ which was followed by the unsharp mask filter^39^ to retrieve the image sharpness. To ensure our analysis stays consistent within the entire data set we created a pore space mask from the dry rock image using watershed segmentation method^40^. Applying the pore space mask on different images removed the rock phase which in turn facilitated the segmentation of oil and water phases using simple thresholding. Labelling^41^ was used to identify each individual connected objects in each image.

## Electronic supplementary material

Supplementary Information
