# Supplementary material for: The Dynamics of Nanoparticle-enhanced Fluid Displacement in Porous Media - A Pore-scale Study
Source: Sci Rep. 2018 Jul 24;8:11148. doi: 10.1038/s41598-018-29569-2 (PMC6057960; doi:10.1038/s41598-018-29569-2)
Supplement: Supplementary file 1 — Supplementary Information [file 41598_2018_29569_MOESM1_ESM.docx]

**Title: The** Dynamics of Nanoparticle-enhanced Fluid Displacement in Porous Media - A Pore-scale Study

**Author List:**

Tannaz Pak^1*^, Nathaly Lopes Archilha^2^, Iara Frangiotti Mantovani^3^, Anderson Camargo Moreira^3^, Ian B. Butler^4^

^1^Teesside University, ^2^Brazilian Synchrotron Light Laboratory (LNLS), Brazilian Center for Research in Energy and Materials (CNPEM), Zip Code 13083-970, Campinas, Sao Paulo, Brazil, ^3^University of Santa Catarina, ^4^University of Edinburgh

**Supplementary Information**

Figure S1: (A) UV scattering response (wavelength: 228 nm) for nanofluids (SiO_2_, 30 nm) at different concentrations. The linear correlation with NP concentration shows these nanofluids are stable. (B) UV scattering response (wavelength: 268 nm) for nanofluids at 0.05 and 0.1 wt% silica concentration. This plot shows the suspensions were stable within the first week (specifically the first two days) after preparation.

Figure S2: Top row: example 2D slices through the reconstructed images of the rock at the four injection steps, Bottom row: 3D renderings show oil phase at the end of the four injection steps.

Figure S3: Example 2D µCT slices showing the emulsion confined in different pores after the 0.12 wt% NP injection. Oil and nanofluid are the bright and dark phases, respectively.

Figure S4: Pore-throat size distribution of three Silurian dolomite samples (D=2.54 cm) extracted from Mercury porosimetry data.

Figure S5: Experimental Set-up

Table 1: Injection conditions for each experiment.

| Injection Step | Flow rate (µL/min) | Pore Volume |
| --- | --- | --- |
| Oil injection | 200 then 500 | >10 |
| Water injection | 10 | 12.25 |
| Nanofluid injection: 0.06 wt% | 10 | 3 |
| Nanofluid injection: 0.12 wt% | 10 | 3 |
